# Potential Drugs and Remedies for the Treatment of COVID-19: a Critical Review

**DOI:** 10.1186/s12575-020-00129-1

**Published:** 2020-07-23

**Authors:** Fatemeh Barati, Mahdi Pouresmaieli, Elena Ekrami, Sahar Asghari, Farzad Ramezani Ziarani, Matin Mamoudifard

**Affiliations:** 1 Department of Industrial and Environmental Biotechnology, National Institute of Genetic Engineering and Biotechnology (NIGEB), Tehran, Iran; 2 Department of Microbiology, School of Medicine, Golestan University of Medical Sciences, Gorgan, Iran

**Keywords:** Coronavirus, Therapeutic compounds, Drugs, Home remedies, Vaccine, Plasma

## Abstract

**Abstract:**

COVID-19 disease with a high rate of contagious and highly nonspecific symptoms, is an infectious disease caused by a newly discovered coronavirus. Most people who fall sick with COVID-19 will experience mild to moderate symptoms such as respiratory symptoms, cough, dyspnea, fever, and viral pneumonia and recover without any special cure. However, some others need special and emergency treatment to get rid of this widespread disease. Till now, there are numbers of proposed novel compounds as well as standards therapeutics agent existed for other conditions seems to have efficacy against the 2019-nCoV. Some which are being tested for MERS-CoV and SARS-CoV are validated that could be also efficient against this new coronavirus. However, there are currently no effective specific antivirals or drug combinations introduced for 2019-nCoV specifically that be supported by high-level evidence. The main purpose of this paper is to review typical and ongoing treatments for coronavirus disease including home remedies, herbal medicine, chemical drugs, plasma therapy, and also vaccinies. In this regards, famous herbal medicines and common chemical drugs which are routinely to be prescribed for patients are introduced. Moreover, a section is assigned to the drug interactions and some outdated drugs which have been proved to be inefficient. We hope that this work could pave the way for researchers to develop faster and more reliable methods for earlier treatment of patients and rescue more people.

**Graphical abstract:**

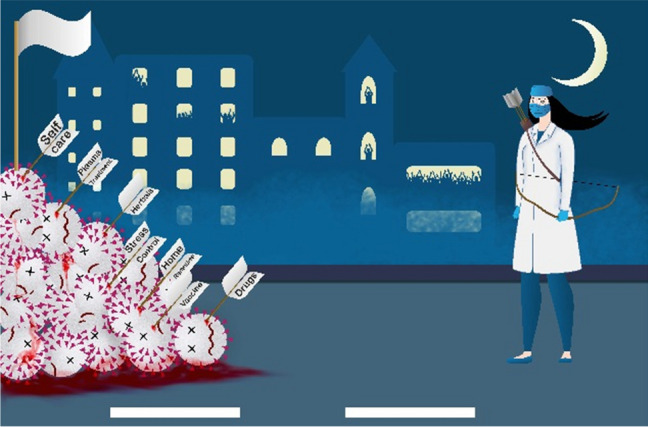

## Introduction

On 30 January 2020 the WHO declared global PHEIC about the epidemic problem of a new coronavirus called 2019 novel coronavirus (2019-nCoV), which was first appeared in Wuhan wet market, Hubei province [1]. This virus genetically is very similar to a bat coronavirus in the subgenus Sarbecovirus [2]. The WHO named the disease as COVID-19 [3] and it spread all over the world rapidly less than 3 month*s. corona*viruses can be classified into four genera (α, β, γ, and δ) and they are detected in a very wide selection of animal species, including humans [4]. The virus that causes the COVID-19 infection belongs to the β coronaviruses family. Since the beginning of the twenty-first century, three coronaviruses crossed the species barrier and causes deadly pneumonia in humans: severe acute respiratory syndrome coronavirus (SARS-CoV) [5], Middle-East respiratory syndrome coronavirus [6], and SARS-CoV-2 [7]. Unlike previous coronaviruses that caused large-scale epidemics such as the Middle East Respiratory Syndrome (MERS) and Severe Acute Respiratory Syndrome (SARS), the transmission rate for COVID-19 is much higher [8]. Due to the high rate of contagious and highly nonspecific symptoms of this disease such as respiratory symptoms, cough, dyspnea, fever, and viral pneumonia [7], the establishment of precise and fast diagnostic tests are urgently required to verify suspected cases, screen patients, and conduct virus surveillance.

The disease is typically confirmed by reverse-transcription polymerase chain reaction (RT-PCR) reverse Real-Time PCR assay (rRT-PCR), which can be carried out using a variety of clinical specimens, including Bronchoalveolar lavage fluid, fibro bronchoscope brush biopsies, sputum, nasal swabs, pharyngeal swabs, feces, or blood [9]. But it has been reported that the sensitivity of RT-PCR might not be enough for the diagnosis of the disease. So the Computed tomography (CT), as a noninvasive imaging approach, can detect certain characteristic manifestations in the lung associated with COVID-19 [7, 10].

The main goal of this paper is to review the newly developed or in developing medicines or remedies for the treatment of COVID-19 disease to help researchers around the world to find advantage and disadvantage of each method and select the best one.

## Coronavirus History

The Nidovirales order, includes Coronaviridae, Roniviridae and Arteriviridae families and Coronaviruses (CoVs) are the greatest group of viruses that relate to this order and Coronaviridae family [11]. Coronaviruses have a single-strand RNA genome with 26 to 32 kilobases in length [12]. There are different hosts for coronaviruses but their special hosts are different types of avian hosts [13]. However, they have also other hosts like different kinds of mammals, including mice, bats, masked palm civets, camels, cats, and dogs [14]. New mammalian hosts are identified for coronaviruses these days and they are rapidly growing up [12]. For example, fatal severe diarrhea syndrome in pigs, associated with an HKU2-related coronavirus of bat source was reported in 2018 [15]. There are different coronaviruses, but the majority of them are associated with mild clinical symptoms [12]. SARS-CoV-2 is a virus from coronaviruses family which causes infection in humans; SARS-CoV, MERS-CoV, and SARS-CoV-2 can cause acute disease, while NL63, HKU1, OC43, and 229E cause mild symptoms [16]. The first time that Severe acute respiratory syndrome (SARS) coronavirus (SARS-CoV) appeared, it was in Guangdong, southern China, in November 2002 and the first time when the Middle East respiratory syndrome (MERS) coronavirus (MERS-CoV) emerged, it was in 2012 in Saudi Arabia [6, 17, 18]. During 2002–03, more than 8000 infected cases by SARS-CoV and more than 774 deaths in 37 countries were confirmed, and MERS-CoV outbreak in September 2012, caused 2494 confirmed cases of infection and 858 mortalities, including 38 fatalities in South Korea [19, 20]. In late December 2019, many infected patients with viral pneumonia were reported, but the point was that all of them were associated with the Huanan seafood wholesale market [21]. So, a new virus which was associated with infecting human was confirmed and provisionally named 2019 novel coronavirus (2019-nCoV), and this virus was detected with the use of next-generation sequencing.

Reported cases with SARS-CoV-2 are now more prevalent, on 28 April 2020, 3,121,118 cases have been reported in 210 countries, and the number of death stands at 216,508 in the world [22] at 20:52 GMT. At present, available data shows that 2019-nCoV spread the infection to the human population from a bat source, but it is unknown if other animal species are associated with this virus and acting as an intermediate host between humans and bat or not.

Full-genome sequencing of 2019-nCoV shows that this virus is genetically similar to bat-SL-CoVZC45 (sequence identity 87・99%; query coverage 99%) and SARS-like betacoronavirus of bat origin, bat-SL-CoVZXC21 (accession number MG772934;23 87・23%; query coverage 98%) [23].

Genetic-wise the 2019-nCoV strains are less similar to SARS-CoV and MERS-CoV (about 79 and 52%, respectively). Samples from Wuhan and China after Sequencing indicated that this virus comes from the subgenus Sarbecovirus. Also, it shows that it is more analogous to bat-SL-CoVZC45 and bat-SL-CoVZXC21, which both are bat-derived coronavirus strains than to other recognized human infecting coronaviruses, which caused the SARS outbreak of 2003 [24]. Coronaviruses have an evolutionary rate of 10^− 4^ nucleotide substitutions per site per year [14]. Their genetic sequence endures mutations at every replication cycle. Due to every fact and information, 2019-nCoV emerged from one source instantaneously. However, as the virus is transmitted to more individuals, arising mutations should be investigated. All the evidence indicates that there is a relationship between bats and the novel coronaviruses, but facts show that there is another animal and it may act as an intermediate.

## Coronavirus Treatment Methods

Most of the COVID-19- infected cases will face mild to moderate respiratory illness and they will recover without requiring special therapies. Elders and those with underlying medical problems such as cardiovascular disease, chronic respiratory disease, diabetes, and cancer are more in danger to develop serious illnesses. At this time, there are no specific treatments or vaccines for COVID-19. However, many ongoing clinical trials are evaluating potential treatments. Herein, we investigated the typical treatments for COVID-19 including Home remedy, Herbal medicine, chemical drugs, plasma therapy, and also vaccines.

### Home Remedy and Herbal Medicine against SARS-CoV-2

Most over-the-counter treatments only cure the symptoms of viral infections but they don’t help the immune system to fight it. Although there is no research to determine what is helpful specifically for this novel virus, the following are some natural modalities one can use to both treat symptoms as well as boost her/his immune system if come down with an illness:

#### Home Remedy

According to statistics WHO, more than 80% of COVID-19 patients should care for themselves at home [25]. So, in this part, some suggestions for treating and prevention of SARS-Cov-2 for Stress reduction**,** self-care, **sore throats, and respiratory congestion and sinuses** are given.

##### Stress Reduction

With the outbreak of coronavirus disease (COVID-19) in 2020, and with increasing numbers of confirmed cases and fatalities, both medical personnel and the public have been experiencing psychological problems, including stress, anxiety and depression [26, 27]. On January 26, 2020, the National Health Commission of China has determined some guideline documents, starting with the notification of principles for emergency psychological crisis therapies for the COVID-19 epidemic [28]. Online mental health services that are used for the COVID-19 epidemic are helping the improvement of Chinese public emergency interventions and eventually could improve the effectiveness and quality of emergency interventions [28]. WHO recommends physical activity 30 min per day for each healthy adults and hour for children per day. Also, the WHO recommends Dancing, skipping rope, some training for muscle strength and online exercise classes for the reduction of stress and boosting the immune system [25].

##### Self-Care

When upper respiratory infections occur, rest and plentiful hydration are helpful. At this time, the first step is drinking enough fluids, some foods including bone broths and homemade vegetables like garlic are helpful too. Garlic is a healthy food that may have some antimicrobial properties. But there is not any strong evidence from the current outbreak that eating garlic has helped people protecting from the COVID-19 (https://www.who.int/emergencies/diseases/novel-coronavirus-2019/advice-for-public/myth-busters). Some of the great herbal teas/hot drinks including ginger, peppermint, eucalyptus, chamomile, and hot water with honey, lemon and cinnamon are helpful for hydration and decreasing the symptoms of the infectious [29].

##### **Sore Throats**

The first step in battling bacterial throat infections is saltwater gargles, which is perfect for loosening mucus and helping fend off. Hot teas and lozenges involving slippery elm are perfect demulcents (to relieve minor pain and inflammation of mucous membranes) for soothing irritated sore throats. A cup of hot water with two tablespoons of honey is an excellent way to soothe and reduce throat inflammation and pain. One of the great helpful treatments for soothing irritated sore throats is peppermint and chamomile tea, other treatments such as teas or infusions made from licorice root and marshmallow root are excellent for soothing demulcents [30]. On April 15, 2020, in Social media, users have been sharing a post with text claiming that gargling with warm water and salt or vinegar will kill the coronavirus (https://www.who.int/emergencies/diseases/novel-coronavirus-2019/advice-for-public/myth-busters). Johns Hopkins Medicine also rejected this claim that gargling with warm saltwater eliminates coronavirus. (https://www.hopkinsmedicine.org/health/conditions-and-diseases/coronavirus/2019-novel-coronavirus-myth-versus-fac) Sore throat is a possible symptom of coronavirus, but it isn’t among the most common, one of the effective home remedies for a sore throat is gargling with warm saline (saltwater) (https://www.webmd.com/cold-and-flu/features/does-gargling-wlth-salt-water-ease-a-sore-throat#1). We were unable to find any official government or medical advisory recommending the use of saline or vinegar to kill the coronavirus.

##### **Respiratory Congestion and Sinuses**

For respiratory congestion, useful things are vaporizers, humidifier, or steam inhalers, and also spending time in steamy baths or showers are helpful. Inhalers and vaporizers can be applied with decongestants or essential oils like peppermint, menthol, eucalyptus or frankincense. Nasal xylitol sprays are very effective, as is nasal irrigation applying a small pot or nasal irrigation bottle. Buffered saline is simple to make, but there are Buffered saline packets in shops too. It removes any irritation to delicate, irritated mucous membranes [31]. ‘Gargle with warm water and salt kills the tonsils microbe and prevents them from leaking into the lungs [32]. There is no official evidence to indicate that these home remedies are effective for eliminating coronaviruses.

#### Herbal Medicine

These days’ herbal medicine plays a major role in the prevention and treatment of many diseases also for the novel coronavirus. Chinese medicine is the pioneer of herbal medicine among all of the countries. Decoctions are suggested for clearing the lung and detoxification by Chinese medicine [33]. Clinical trials have demonstrated Chloroquine was proved to be effective in the control and treatment of SARS-CoV2 [34]. There was wide usage of traditional Chinese medicine through the last SARS-COV outbreak and it is currently being applied in China. The five most famous applied herbs were *Astragali Radix (Huangqi)*, *Saposhnikoviae Radix (Fangfeng), Glycyrrhizae Radix Et Rhizoma (Gancio), Atractylodis Macrocephalae Rhizoma (Baizhu),* and *Lonicerae Japonicae Flo* [35].

However, available data on effective herbal medicine for infected cases with SARS-CoV-2 were inadequate, and hence further studies are required [36]. In this review, other Chinese medicinal plants can be found in Table 1 [35, 37, 38] and herbal plants public beliefs are figured out in Table 2, among all kinds of herbal plants in the world. Table 3 shows some additional herbal drugs which are used for immunity booster all over the world properly by various communities.

**Table 1:** The Chinese herbal plant that used for treating for SARS-CoV-2

No	Latin herbal plant name	Usage
1	*Forsythia Fructus*	Antipyretic-detoxifying
2	*Lonicerae japonica flos*	
3	*Rhizoma fagopyri cymosi*	
4	*Houttuynia herba*	
5	*Hoveniae dulcis semen*	
6	*Mori cortex*	Antitussive antiasthmatics
7	*Farfarae flos*	
8	*Eriobotrya folium*	
9	*Lepidii semen descurainiae semen*	
10	*Ardisiae japonicae herba*	
11	*Asteris radix etrhizoma*	
12	*Ginkgo semen*

**Table 2:** Public beliefs about herbal plants. Is it effective or not?

No	Herbal plant name	Effective	Ineffective
1	*Licorice* [37]	*	
2	*Cardamom*	Unknown	
3	*Peppermint* [39]	*	
4	*Ginger* [39]	*	
5	*Eucalyptus* [39]	*	
6	*Chamomile* [39]	*	
7	*Hot water with lemon* [39]	*	
8	*Honey* [39]	*	
9	*Cinnamon* [39]	*	
10	*Banana* [40]		*
11	*Onion* [41]	*

**Table 3:** Some herbal drugs which are used in countries for immunity booster

No	Herbal plant name	No	Herbal plant name
1	*Honey, Piper nigrum and Curcuma longa*	8	*Dried astragalus root* (https://www.healthline.com/health/food-nutrition/immune-system-bitters-recipe#Recipe-for-an-immune-boosting-bitters)
2	*Dried angelica root* (https://www.healthline.com/health/food-nutrition/immune-system-bitters-recipe#Recipe-for-an-immune-boosting-bitters)	9	*Dried chamomile* (https://www.healthline.com/health/food-nutrition/immune-system-bitters-recipe#Recipe-for-an-immune-boosting-bitters)
3	*Dried ginger* [42]	10	*Dried orange peel* (https://www.healthline.com/health/food-nutrition/immune-system-bitters-recipe#Recipe-for-an-immune-boosting-bitters)
4	*Cinnamon stick* [42]	11	*Cardamom seeds* (https://www.healthline.com/health/food-nutrition/immune-system-bitters-recipe#Recipe-for-an-immune-boosting-bitters)
5	*Moringa* (https://www.medlife.com/blog/boost-immunity-5-single-herb-supplements/)	12	*Amalaki* (https://www.medlife.com/blog/boost-immunity-5-single-herb-supplements/)
6	*Neem herbs* (https://www.medlife.com/blog/boost-immunity-5-single-herb-supplements/)	13	*Licorice* (https://www.medlife.com/blog/boost-immunity-5-single-herb-supplements/)
7	*Onion* [41]	14	*Honey* [40]

### Chemical Drugs

Nowadays, scientists are looking for discovering drug agents or complexes in order to treat COVID-19. These drugs usually used as antiviral agents for other diseases and their function about COVID-19 is unrevealed, which needs more prolonged studies. Herein, we will mention various drugs used for viral disease treatment which is also showing inhibitory effect, etc. in COVID-19 infection.

#### Favipiravir

Favipiravir is a drug approved for the treatment of influenza in China. The action mechanism of Favipiravir is to inhibit RNA-dependent RNA polymerase. In addition to the action against the influenza virus, this antiviral drug can inhibit the replication of flavi, alpha, filo, bunya, and other RNA viruses [42]. Following the entry to the cells, Favipiravir is transformed into an active form by becoming phosphoribosylated (favipiravir-RTP) and will recognize viral RNA polymerase, inhibiting its activity [43]. So, Favipiravir may have the potential to act against SARS-CoV-2; researches showed that Favipiravir independently associates with more active viral clearance and higher improvement rates in the chest imaging and has a positive impact on treating patients with COVID-19 positive tests [44]. In a study in Wuhan, Favipiravir was administered to 200 patients, and their test results were appeared negative after a relatively short time. Also, the symptoms of pneumonia were significantly reduced. In another study in Wuhan, showed that the patient treated with Favipiravir recovered from fever after an average of 2.5 days, compared to 4.2 days of other patients [45]. Another study showed Favipiravir compared to Lopinavir (LPV)/ritonavir (RTV) associated with shorter time-to-viral-clearance (median 4 versus 11 days, *P* < .001) and significant improvement in chest imaging (improvement rate 91.43% versus 62.22%) [44].

#### Chloroquine and Hydroxychloroquine

Chloroquine (CQ) is a drug that widely used against malaria and in 2006 was observed this drug has an antiviral potential [46]. The function mechanism of Chloroquine is increasing the endosomal pH. With a pH higher than what is required for virus/cell fusion, the infection is blocked. This interferes with glycoproteins of cellular receptors of sars-cov-2 which bind to their targets [47]. Therefore, Chloroquine can inhibit a pre-entry step of the viral cycle via interfering with the binding of viral particles to their cellular cell surface receptor [48]. In one study carried out by a Chinese researchers group about the efficiency of chloroquine in in-vitro studies, using Vero E6 cells infected by SARS-CoV-2 at a multiplicity of infection (MOI) of 0.05, Chloroquine was highly effective in decreasing viral replication and it can block SARS-CoV-2 infection at low concentration (half-maximal effective concentration (EC50) of 1.13 μM and a half-cytotoxic concentration (CC50) larger than 100 μM) [49]. Another article indicated that Chloroquine can inhibit in-vitro replication of HCoV-229E in epithelial lung cell cultures [50, 51] and also can be effective against Middle East respiratory syndrome coronavirus (MERS-CoV) in-vitro [52].

Hydroxychloroquine (HCQ) with a very similar chemical structure to Chloroquine is one of the disease-modifying antirheumatic drugs that use for healing many rheumatic diseases and also demonstrate a strong immunomodulatory capacity which prevents inflammation flare-ups and organ damage [53]. Both Chloroquine and Hydroxychloroquine can raise the intracellular pH and inhibit the fusion process between viruses and endosomes [47]. They can also inhibit nucleic acid replication, glycosylation of viral proteins, virus assembly, new virus particle transport, virus release, and other processes to achieve its antiviral outcomes [54]. In one case, the pharmacological activity of chloroquine and hydroxychloroquine was checked out, the Vero cells were infected with SARS-CoV-2 and Hydroxychloroquine (EC50 = 0.72 μM) was found to be more potent than chloroquine (EC50 = 5.47 μM) in-vitro because of smaller EC value [55]. On 4 June 2020, the UK Medicines and Healthcare Products Regulatory Agency (MHRA) said that ‘We have concluded that there is no beneficial effect of hydroxychloroquine in patients hospitalized with COVID-19. We have therefore decided to stop enrolling participants to the hydroxychloroquine arm of the RECOVERY Trial with immediate effect. We are now releasing the preliminary results as they have important implications for patient care and public health.

‘A total of 1542 patients were randomized to Hydroxychloroquine and compared with 3132 patients randomized to usual care alone. There was no significant difference in the primary endpoint of 28-day mortality (25.7% Hydroxychloroquine vs. 23.5% usual care; hazard ratio 1.11 [95% confidence interval 0.98–1.26]; *p* = 0.10). There was also no evidence of beneficial effects on hospital stay duration or other outcomes. Full results will be made available as soon as possible [56].

#### Remdesivir

Remdesivir is an adenosine nucleotide analog that is similar to tenofovir alafenamide - a nucleotide analog of adenosine 5-monophosphate - with antiviral activity against hepatitis B virus, HIV [55], filoviruses, paramyxoviruses, and pathogenic coronaviruses, like SARS-CoV and MERS-CoV [57]. In in-vitro research, the EC_50_ of Remdesivir against SARS-COV-2 in Vero E6 cells was 0.77 μM and the EC_90_ was 1.76 μM [58]. The symptoms of a COVID-19 positive patient in Washington, USA treated with Remdesivir improved and no noticeable side effects were observed. Finally, 13 days after treatment with Remdesivir the result of real-time RT-PCR analysis from the oropharyngeal swab was negative for SARS-CoV-2 [59].

#### Ribavirin

Ribavirin is a nucleotides derivative competing with physiological nucleotide for RdRp active site [60, 61]. Through the outbreak of SARS in Hong Kong, ribavirin was widely used for patients with or without the simultaneous use of steroids [62]. The EC_50_ against COVID-19 for Ribavirin determined at 109.5 μM [63]. Based on previous studies about SARS and MERS, the combination of ribavirin and IFN-β, decreases the viral replication and disease severity in animal models [64]. Due to unfavorable reactions, the proper dose of ribavirin in the clinical application should be given carefully.

#### Losartan

Losartan is an AT1 antagonist which its function is specific and competitive for decreasing of the responses to AngII. This drug is a common anti-hypertensive agent that is presently suggested for the regulation of high blood pressure, particularly those cases that are prone to diabetic nephropathies. Losartan causes the release of aldosterone that is the physiological effects of AngII. Then plasma renin activity increases due to the absence of AngII and some biochemical events occur such as AngI to AngII (by ACE), converting angiotensinogen to AngI, vasoconstriction and aldosterone release (by AngII). The presence of aldosterone leads to recollecting sodium from the kidney which finally increases the blood pressure. Thus, losartan can reduce the blood pressure. Accordingly, losartan is a selective antagonist of the AT1 receptor which employs an inhibitory effect on the ACE-AngII-AT1 axis in the Renin-Angiotensin-Aldosterone System (RAAS). So, it may be an effective drug to protect from lung damage induced by coronavirus [65]. Based on earlier laboratory researches, losartan also reduces the synthesis of TGF-1β and Poly (ADP-ribose) polymerase (PARP). Therefore, it can prevent or control persistent fibrotic diseases such as cardiac hypertrophy and asthma in addition to hypertension. Moreover, apart from direct anti-hypertensive outcomes of losartan, it provides a notable decrease of platelet aggregation by ristocetin and reduction of hematocrit with hemoglobin, following administration in newly diagnosed hypertensive patients which is suggestive for applying losartan for thrombosis and atherosclerosis as well. Other lateral functions of losartan considered to be the immunomodulatory role and significantly regulating IFN-γ, IL-6, IL-17F, and IL-22 cytokines in peripheral blood mononuclear cells (PBMCs) of rheumatoid arthritis patients.

Finally, an earlier hypothesis illustrated that losartan as an angiotensin receptor 1 (AT1R) blocker in the RAS pathway could be useful for patients infected by COVID-19 who experience pneumonia [66].

The role of the Renin-Angiotensin-Aldosterone System (RAAS) in COVID-19 infection is a hot topic of discussion. RAAS inhibitors, such as Angiotensin Converting Enzyme (ACE) inhibitors and Angiotensin II receptor blockers (ARBs), which are used to treatment of cardiovascular diseases, have been resulted in increased cell surface levels of ACE2. ACE2 is the host receptor for COVID-19 which discovered in Wuhan, China in December 2019. Angiotensin receptor blockers (i.e., AT1R), like losartan, olmesartan and valsartan can inhibit Ang II-mediated vasoconstriction. Besides, aldosterone inhibitors (e.g., spironolactone, eplerenone, amiloride) control vascular tone by affecting sodium channels for sodium reabsorption in the kidneys to maintain blood volume [67]. Early experiments indicated that the interaction of the spike protein of the virus with the ACE2 leads to a decrease in ACE2 levels in cells, which causes lung damage. So the block of other available receptors is suggested, e.g. AT1R blockers (Angiotensin II receptor blocker) such as losartan, can help in curing COVID-19. However, this approach also needs more clinical patient records [68].

#### Interferon

IFNs are a group of cytokines, which communicate between cells against pathogens and have a critical role in the immune system, such as activating natural killer (NK) cells and macrophages. There are three classes of IFNs: I (such as IFN-α and –β), II (IFN-γ), and III, all of which play roles against viral infections [69].

Type-I interferon (IFN-I) activates intracellular pathogen defense and influences the development of innate immunity and adaptive immunity. The DNA sensor cyclic GMP–AMP synthase (cGAS) and the stimulator of interferon genes (STING) can control transcription of many inflammatory mediators, including type I and type III interferons [70].

Reports showed that the administration of moxifloxacin, lopinavir, and interferon to non-ICU patients and the addition of methylprednisolone to the above treatment for ICU patients resulted in 26 patients being discharged from ICU and 16 patients being discharged from hospital [71].

Signal transducer and activator of transcription 1 (STAT1), a key protein in the interferon-mediated immune response, is antagonized by the virus. This can indicate the increased response threshold of immune cells to IFNs during CoV infections. Differences in the primary immune responses due to the interferon -depending on the age- lead to the various mortality rates. The increase in these rates in the elderly can be explained through the higher threshold of interferon-mediated immune responses. According to this, earlier induction of interferons in children and their less developed immune system is the main factor for their near to zero lethality rate [69].

Many researchers reported that their data about the identified gene expression signatures of viral invasion and type I interferon (IFN-I) responses is the key characterizing factor for the diagnosis of the COVID-19 infection stage in humans. The relationship between viral load/IFN levels and disease severity in COVID-19 infection marked the key difference in the pathogenesis of this new from the previous coronavirus such as SARS-CoV infections [72].

#### Lopinavir – Ritonavir

Three anti-HIV drugs, ritonavir, lopinavir, and darunavir, might have a therapeutic impact on coronavirus disease 2019 (COVID-19). It is implied that the therapeutic effect of ritonavir and lopinavir on COVID-19 may be largely due to their inhibitory impact on CEP_C30 (coronavirus endopeptidase C30), while ritonavir may have greater ability; the inhibitory impact of darunavir on SARS-CoV-2 and its potential therapeutic influence may be largely due to its inhibitory effect on PLVP(papain-like viral protease) [73].

Lopinavir is metabolized by cytochrome P4503A (CYP3A) isoenzyme in the liver. It is always used with ritonavir to reduce the dose of lopinavir and enhance the plasma levels of lopinavir as ritonavir inhibits CYP3A isoenzyme. Lopinavir and ritonavir are antiretroviral protease inhibitors used in combination as a second-line drug for the treatment of HIV-1 infection in children and grown-ups through the fewer side effects. A few historical control studies or case reports show the effectiveness of the combination of lopinavir/ritonavir against SARS-CoV and MERS-CoV infections. The reduction of steroid consumption and nosocomial infections was seen in patients initially treated with lopinavir/ritonavir. Also, results indicate decreasing viral load and enhancing peripheral lymphocyte count. Findings from in vitro and clinical studies, together with the availability and safety profiles of lopinavir/ritonavir and interferon beta-1b (IFN-β1b) suggest that the combination of these agents has potential efficacy for the treatment of patients with MERS-CoV [74–76].

The question of whether the earlier lopinavir-ritonavir approach in COVID-19 could have clinical benefit is an important one that needs further study. A new report showed that the average span of viral shedding in COVID-19 was 20 days in patients with severe illness and could be as long as 37 days. There isn’t any proved evidence that lopinavir-ritonavir showed a significant antiviral effect. The reason for this deficit is suboptimal methods of sampling using in the current trial. In brief, lopinavir-ritonavir treatment did not significantly improve clinical development or reduce-throat viral RNA detectability in patients with serious COVID-19 [77].

#### Actemra – Tocilizumab

Neutralizing antibodies against nCoV 19 surface antigens block virus entry, of which S protein considered a good candidate. These monoclonal neutralizing antibodies provide passive immunity in the time of exposure like palivizumab as a putative model that has been applied for the prevention of RSV infection. Anti-inflammatory antibodies like anti IL6 receptor (Tocilizumab) and anti ITGA4 (Natalizumab) may repress inflammation and cellular extravasation [78].

Based on similarities between the new virus (SARS-CoV-2) and SARS I, COVID-19 decreases a key enzyme which plays a role in the regulation of the water and salt concentration in the blood, so it could contribute to pneumonia seen in severe cases.[78].

Tocilizumab is an efficient remedy in patients of COVID-19 with severe symptoms, which provided a new therapeutic approach for this fatal infectious disease. By using this drug after a few days, the fever eliminated and all other clinical symptoms improved significantly. Oxygen intake for 75%of patients decreased and one patient needs no oxygen therapy. CT scans revealed that the lung lesion opacity absorbed in 19 patients (90.5%) and totally, other blood factors improved [79].

As during previous pandemics (SARS and MERS), corticosteroids are not routinely recommended and might exacerbate COVID-19-associated lung injury. However, in hyper inflammation, immunosuppression is likely to be beneficial. Re-analysis of data from a phase 3 randomized controlled trial of IL-1 blockade (anakinra) in sepsis, showed meaningful survival impacts in patients with hyper inflammation, without extra side effects. A default controlled trial of tocilizumab (licensed for cytokine release syndrome), has been approved in patients with COVID-19 pneumonia and raised IL-6 in China (ChiCTR2000029765) [80]. Also, disruption of IL-6 signaling with anti-IL-6 receptor monoclonal antibody, tocilizumab, showed beneficial effects in treating Chinese COVID-19 patients with cytokine storm syndrome [81].

Studies have shown that IL-6 levels significantly associated with the severity of COVID-19, C-reactive protein (CRP), lactate dehydrogenase (LDH), and D-dimer levels and T cell numbers, and it has been recommended that Tocilizumab, with its inhibitory effect on IL-6, may be effective in the treatment of COVID-19 [71].

#### Vitamin D

Vitamin D has many mechanisms that reduce the risk of microbial infection and death. Vitamin D with enhance cellular innate immunity partly through the induction of antimicrobial peptides, including human cathelicidin, LL-37, by 1,25-dihydroxy vitamin D and defensins that has antimicrobial activity against Gram-positive and Gram-negative bacteria, enveloped and nonenveloped viruses, and fungi. It is known that COVID-19 infection is associated with the increasing production of pro-inflammatory cytokines, C-reactive protein, increase risk of pneumonia, acute respiratory distress syndrome, and heart failure. (Evidence that Vitamin D Supplementation Could Reduce the Risk of Influenza and COVID-19 Infections and deaths). Vitamin D also deregulates the inflammatory response, especially of the renin-angiotensin system, which is characteristic of COVID-19, and degree of overactivation is associated with poorer prognosis. Vitamin D is known to interact with a protein in this pathway—angiotensin-converting enzyme 2 (ACE2)—which is also exploited by SARS-CoV-2 as an entry receptor. Downregulates expression of ACE2, vitamin D promotes the expression of this gene [82, 83].

But so far there is no clear evidence for that vitamin D can prevent or treat COVID-19 infection and should be further studied before it is recommended to patients.

#### Zinc

Zinc is known to be important for immune function and has a role in antibody and white blood cell production. Zinc increase pro-inflammatory cytokine (IL-1, IL-6, and TNF alpha) concentrations and decreases the production of antibodies, while zinc supplementation has been shown to increase the ability of polymorphonuclear cells to fight infection. It is show that when the intracellular Zn^2+^ concentration is raised, can be interfere with proteolytic processing of polyproteins in many RNA viruses like coronavirus family. All coronaviruses are unified in requiring a RNA-dependent RNA polymerase (RdRp) that is a core enzyme in their RNA-synthesizing machinery. Increased Zn^2+^ levels directly inhibit isolated RdRp complexes and purified. Zinc supplementation can benefit patients with lower respiratory tract infections such as COVID-19. Because of its role in immune function and potential to decrease coronavirus replication, zinc is currently being investigated for prophylaxis and treatment of patients with COVID-19 [84–86].

#### Drug Interactions

Although using multiple drugs at the same time may lead to a synergistic impact, it’s also possible to result in drug-drug interactions with dangerous or even lethal effects. Therefore, it’s necessary to study them to prevent irreparable results. Tables 4 and 5 showed interactions between common drugs used for COVID-19. Supplementary information will be achieved through extra analyzes over time.

**Table 4:**
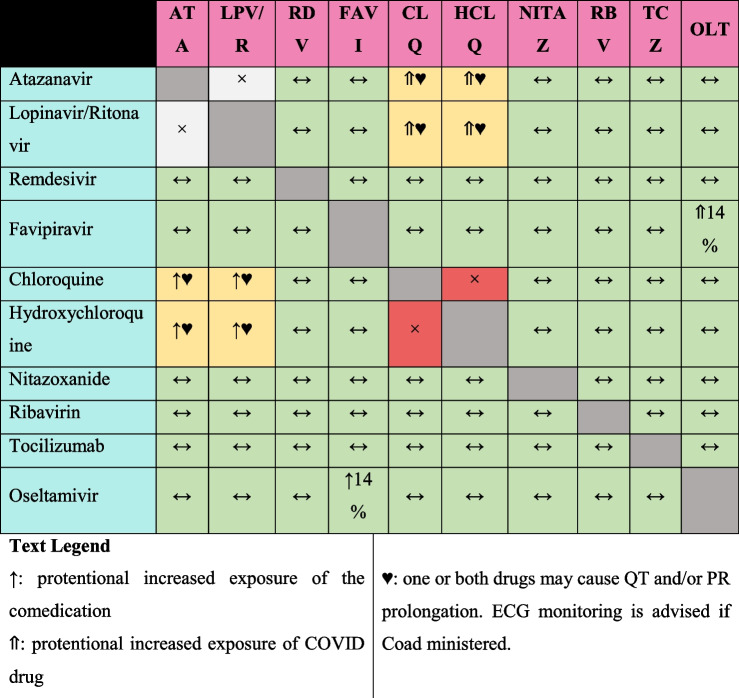
Interaction between regular usage drugs (http://www.covid19-druginteractions.org)

**Text Legend**

↑: protentional increased exposure of the comedication

⇑: protentional increased exposure of COVID drug

↔: no significant effect

♥: one or both drugs may cause QT and/or PR prolongation. ECG monitoring is advised if Coad ministered

Numbers refer to an increase or decrease in AUC as observed in drug-drug infraction studies

**Table 5:** Interaction between drugs [87]

Therapy	Specific interaction
Ribavirin	-Anticoagulants Warfarin
Lopinavir/Ritonavir	-Anticoagulants Apixaban Rivaroxaban -Antiplatelets Clopidogrel Ticagrelor -Statin Atorvastatin Rosuvastatin Lovastatin Simvastatin -Antiarrhythmics QT-prolonging medication Digoxin
Chloroquine/Hydroxychloroquine	-Beta-blockers Metoprolol, carvedilol, propranolol, labetalol -Antiarrhythmics QT-prolonging agents Digoxin

Additionally, it’s noted that Hydroxychloroquine consumption is better than chloroquine (during long-term use) due to its fewer side effects. Besides, it’s probable to use a higher daily dose within fewer drug-drug interactions [88].

### Convalescent Plasma as a Potential Therapy for COVID-19

As it is mentioned before, up to now, there is not any special therapy to be effective for reducing COVID-19 infection. However, there are some supportive treatments, like oxygen supply in mild patients and extracorporeal membrane oxygenation for the critical cases. In SARS-infected cases to enhance the survival rate of patients in cases whose state deteriorated, the last resort in treatment was applying immunoglobulins or convalescent plasma despite therapy with pulsed methylprednisolone. Besides, many different studies indicated a shorter hospital stay and less fatality in patients cured with convalescent plasma [89–91]. In 2014, WHO suggested the use of convalescent plasma acquired from Ebola virus-infected cases who had recovered as an empirical treatment through prevalence [92]. In 2015, a protocol for the use of convalescent plasma in the remedy of Middle East respiratory syndrome coronavirus was appointed [93]. In a study, Hung and colleagues indicated that in the influenza A H1N1 (H1N1pdm09) virus-infected cases in 2009, there was a great decrease in the relative risk of mortality for those cases treated with convalescent plasma [94]. Besides, in a subgroup analysis, after convalescent plasma therapy viral load was lower on days 3, 5, and 7 after intensive care unit admission. There were not any adverse events [95]. One of the results for indicating the efficacy of convalescent plasma treatment is that the antibodies from these kinds of plasma might repress viremia. Schoofs et al. described that 3BNC117-mediated immunotherapy, which is a wide neutralizing antibody to HIV-1, increases host humoral immunity to HIV-1 [96].. Moreover, an in vivo test indicated that the effects of this antibody were not only blocking new infection and viral clearance but also involves fast clearance of infected cells [97]. In the first week of infection, Viraemia peaks occur in viral infections. By days 10–14 and through virus clearance, the patient usually arises a primary immune response [91]. So, theoretically, it looks like that it should be more useful to administer the convalescent plasma at the primary stage of disease [98]. However, other therapies might affect the connection between antibody level and convalescent plasma, involving antiviral drugs, intravenous immunoglobulin, and steroids [99]. WHO recommended the main focus on infection prevention, infected detection and monitoring, and supportive treatment for COVID-19. However, there is not any special anti-SARS-CoV-2 treatment recommended due to the lack of evidence. There is a recommendation in a Comment in *The Lancet*, which emphasizes that systematic corticosteroids should not be given typically for the remedy of COVID-19 [100]. Evidence indicates that convalescent plasma from patients who have recovered from viral infections can be applied as a therapy without the occurrence of severe harmful events. So, it might be beneficial to test the efficacy and safety of convalescent plasma transfusion in SARS-CoV-2-infected cases [101]. There are more than 30,000 recovered COVID-19 patients and they are the perfect source of convalescent plasma for health care providers. China National Biotec Group Co has claimed that 10 severely ill patients receiving this convalescent plasma treatment showed better oxygenation and less inflammation and viral load [102]. In one study Chenguang Shen et al., [103] used the plasma therapy method for patients.

Patients with confirmed COVID-19 were allowed to receive convalescent plasma treatment.

Donors of plasma were 5 patients who were recovered from SARS-CoV-2 infection and they were the ages of 18 and 60 years and their convalescent plasma after written informed consent was obtained. Donors were tested negative for SARS-CoV-2 and were tested negative for other respiratory viruses, also for hepatitis B virus, hepatitis C virus, syphilis, and HIV at the time of blood donation. Five patients (age range, 36–73 years; 2 women) were treated with convalescent plasma. None of them were smokers, and 4 of 5 had no preexisting medical conditions. All 5 had received different antiviral steroids and factors (Table 6).

**Table 6:** Clinical Characteristics of SARS-CoV-2-Infected Patients Who Received Convalescent Plasma

	Patient				
	1	2	3	4	5
Sex	Male	Male	Female	Female	Male
Age, y	70s	60s	50s	30s	60s
Weight, kg	55	85	60	41.5	87
Smoking	No	No	No	No	No
Blood type	B	B	B	A	B
Coexisting chronic disease	None	Hypertension; mitral Insufficiency	None	None	None
Disease presentation and course					
Estimated incubation period, d^a^	1	7	3	7	15
Interval between symptom onset and admission, d	2	4	2	2	3
Interval between admission and plasma transfusion, d	22	10	20	19	20
Complications prior to plasma transfusion	Bacterial pneumonia; Severe ARDS; MODS	Bacterial pneumonia; Fungal pneumonia; severe ARDS; myocardial damage	severe ARDS	severe ARDS	severe ARDS
Most severe disease classification	Critical	Critical	Critical	Critical	Critical
Treatments					
Steroids	Methylprednisolone	Methylprednisolone	Methylprednisolone	Methylprednisolone	Methylprednisolone
Antivirals	Lopinavir/ritonavir; Interferon alfa-1b; favipiravir	Lopinavir/ritonavir; Arbidol; darunavir	Lopinavir/ritonavir; Interferon alfa-1b	Interferon alfa-1b; favipiravir	Lopinavir/ritonavir; Interferon alfa-1b

Results indicated that all 5 patients who were receiving mechanical ventilation at the time of transfusion, and 3 patients (patients 3, 4, and 5) were weaned from mechanical ventilation. Patient 2 was receiving ECMO at the time of plasma treatment but did not require ECMO on day 5 after transfusion. Patients 3, 4, and 5 were discharged from the hospital (length of stay: 53, 51, and 55 days, respectively). As of March 25, 2020, patients, 1 and 2 remained hospitalized, with lengths of stay of 37 days each. The results are indicated in Table 7.

**Table 7:** Comparison of Viral Load, Clinical Indexes, and Laboratory Results Before and After Convalescent Plasma Transfusion (continued)

	Patient				
	1	2	3	4	5
IL-6, pg/ml (normal range, 0–7)					
Before transfusion	70.5	438.2	63.9	79.1	87.8
Day 1 posttransfusion	74.9	NT	118.5	39.3	NT
Day 3 posttransfusion	34.5	1045.0	67.0	25.8	797.9
Day 5 posttransfusion	24.1	334.1	590.5	NT	NT
Day 7 posttransfusion	30.8	29.8	174.3	34.0	69.9
Day 12 posttransfusion	6.1	31.8	NT	2.7	54.9
Length of hospital stay, d	Remains hospitalized	Remains hospitalized	53	51	55
Current status as of March 25,2020	Stable, still receiving mechanical ventilation	Stable, still receiving mechanical ventilation	Discharged home	Discharged home	Discharged home

The plasma treatment project as a complementary therapy method initiated on 26 March by collecting plasma from patients who have been recovered from coronavirus to inject to COVID19-infected cases in Iran [104] but the result is not available yet.

In New York and Houston, pints of straw-colored convalescent plasma have been applied for five U.S. coronavirus patients. Hundreds more there and across the nation are set to follow (https://www.newsday.com/news/health/coronavirus/coronavirus-plasma-antibodies-treatments-1.44087471) But the results are not available yet.

Also on April 26, 2020, Scientists from Stony Brook University’s Renaissance School of Medicine, Northwell Health’s Feinstein Institutes for Medical Research and Catholic Health Services started searching to clarify if the antibodies in plasma from recovering COVID-19-infected cases can help stop the infection in people who are still sick or not. Dr. Jason M. Golbin, Catholic Health’s senior vice president and chief quality officer, confessed he is “cautiously optimistic” about this remedy but concedes it will be months before they can define if it’s effective and safe. Up to now, Catholic Health has transmitted 140 convalescent plasma transfusions at its six Long Island hospitals (https://news.sky.com/story/coronavirus-plasma-treatment-for-covid-19-patients-to-be-trialled-11978522).

On Saturday 25 April 2020, Health Secretary Matt Hancock donates to the trial of COVID-19 plasma treatment in the UK [105].

COVID-19 needs quick development of successful medicinal treatment modalities. Convalescent plasma may be one of them. However, there is no evidence to indicate that if this method is good enough or not.

### Under Development Vaccines for SARS-Cov2 Treatment

First, it should be noted that this review has been done by studying the information which was available on websites and literature for public view. Mostly, vaccines that are under development for this virus are made by three strategies; whole vaccines, subunit vaccines, and nucleic acid vaccines.

Whole vaccines: These are Live-attenuated or inactive whole virus, which is the classic strategy for designing a vaccine. The problem with this type is that the virus may go back to the active state and cause infection. Also, it can cause immune potentiation [106].

Johnson & Johnson is working on one that is similar to their platform which was used for the Ebola vaccine; they are using Janssen’s AdVac® adenoviral vector and manufacturing will occur in their PER.C6® cell line technology. This platform is also a candidate for Zika and HIV vaccine. It is at a preclinical stage and is estimated to enter the market next year [107–109].

The University of Hong Kong is working on a live influenza vaccine that expresses SARS-CoV-2 proteins. It is a Modified nasal spray influenza vaccine, with the surface antigen of SARS-CoV-2 and can prevent both influenza and coronavirus. This one is also at a preclinical stage, and it should be tested on animals for months and after that, it should endure at least 1 year of clinical trials on humans [110].

a “codon deoptimization” technology has been developed by Codagenix to attenuate viruses and they are currently exploring strategies for SARA-CoV-2 vaccine development [111].

Chinese Centre for Disease Control and Prevention (CDC) is working on an inactivated virus vaccine, although it is not verified yet. It needs at least 1 month for development and even after that, two or 3 years should pass until it is ready for the market [112, 113].

Subunit vaccines: these use a subunit of the virus which can trigger an immune response in the body. This type can also cause immune potentiation like whole virus vaccines [106].

the University of Queensland is synthesizing viral surface proteins, under funding from the Coalition for Epidemic Preparedness (CEPI), in the purpose of presenting them more easily to the immune system. The technology of use is Rapid Response, a ‘Molecular clamp’ vaccine platform in which the body is misled to generate antibodies by adding genes of viral proteins. The process is now in the preclinical state, estimated to be on the market 6 months after this news went public [112, 113].

By the recombinant expression of the S-protein, Novavax has developed and produced immunogenic virus-like Nanoparticles. It is at the preclinical stage and it is estimated that it will be available for the market 3 months after the announcement [114].

Clover Biopharmaceuticals has a subunit vaccine under development. It is a highly purified recombinant SARS-CoV-2 S protein subunit-trimer vaccine (S-Trimer) and they produced it by using their Trimer-Tag© technology. They have announced that it is now at the preclinical phase, but it is not clear when it will enter the market [115].

Texas Children’s Hospital Center for Vaccine Development at Baylor College of Medicine has developed and tested a subunit vaccine which concludes only the receptor-binding domain (RBD) of the SARS-CoV S-protein. This vaccine elicits high levels of protective immunity on the homologous virus challenge. Also, it can minimize host Immuno potentiation [106].

Also reported by pang et al. [111] GeoVax—BravoVax are working on a “Modified Vaccinia Ankara—Virus-Like Particles (MVA-VLP) vaccine platform”.

Nucleic Acid Vaccines: immunizing with DNA showed promising results in mice in 1993 against influenza, but for decades, it has not progressed for human’s benefit. But More recently, improvements have been shown, especially under critical conditions that humans are facing now due to SARS-CoV-2 pandemic, these efforts could lead to the first licensed human nucleic acid vaccine [101].

Inovio Pharmaceuticals is working on a DNA vaccine. It is an INO-4800-DNA based vaccine, which means that the DNA is synthesized in a lab and does not require an actual virus sample. It will be tested on humans a few months later [116, 117].

While inovio works on DNA vaccine, Moderna Therapeutics, Curevac, and Shanghai East Hospital (Tongji University)—Stermirna Therapeutics, are exploring RNA vaccine platforms. The information available to the public indicates that Moderna is using the Messenger RNA vaccine while two latter are working on mRNA technology. Moderna has a few months to start clinical trials, but much longer is needed so it would be ready for full testing. Others are in the preclinical phase [117–119].

There are few other vaccines under development in which their method and platform are either not available or it cannot be categorized in the mentioned groups. The University of Saskatchewan (VIDO InterVac) has a vaccine at the preclinical phase, which is enduring animal testing and is estimated to be ready for human trials next year (https://www.sciencealert.com/oxford-university-has-just-launched-a-human-trial-of-a-potential-covid-19-vaccine).

News about producing vaccine till 24 April 2020, indicates that even Oxford University has started a human trial of a coronavirus vaccine, with the daunting goal of producing a successful jab available to the public later this year. The volunteers are aged between 18 and 55 and they are in good health and they tested negative for COVID-19 and are not pregnant or breastfeeding (https://www.nytimes.com/2020/04/27/world/europe/coronavirus-vaccine-update-oxford.html). The Oxford researchers now say that with an urgent approval from regulators, the first few million doses of Oxford vaccine could be available by September if it demonstrated to be efficient (https://www.businessinsider.com/india-serum-institute-millions-oxford-university-vaccine-before-approval-2020-4). And The Serum Institute of India declares it will start producing an experimental COVID-19 vaccine while the vaccine is still being tested. Serum Institute CEO Adar Poonawalla declares that “The decision — at our own risk and cost — has been solely taken to get a jump-start on producing vaccine” (https://www.businessinsider.com/india-serum-institute-millions-oxford-university-vaccine-before-approval-2020-4).

And finally, Vir Biotechnology is using whole-genome CRISPR based screening to produce Anti-coronavirus monoclonal antibodies [113].

There are more than 100 research plans around the world to find a vaccine - claimed by the United Nations as the only way back to “normality” - seven are presently in clinical trials, according to the Tropical Medicine and London School of Hygiene. These kinds of trials are already underway in the United States and China and will start at the end of this month in Germany (https://www.nytimes.com/2020/04/08/health/coronavirus-vaccines.html).

#### Monoclonal Antibody

Passive antibody therapy can be a helpful technique to restrict COVID-19 epidemics. Passive antibodies can recognize epitope regions on the foreign virus particle. So they can decrease the disease severity and also the virus replication rate which could be cloned and expressed in suitable expression systems such as mammalian, yeast, or plant. Therefore, recombinant monoclonal antibodies could be tested against SARS-CoV-2 [120]. CoV infections begin with the interaction of the receptor-binding domain located in the S protein and target receptor on the host cell surface such as Angiotensin-converting enzyme 2 (ACE2) [121]. So the specific neutralizing monoclonal antibodies against receptor-binding domain (RBD) in spike protein or specific antibody that binds to ACE2 could efficiently block the virus entry to the cell. In a study, it is shown that monoclonal antibodies can neutralize SARS-CoV, S1, and S2 protein and inhibit syncytia formation between cells expressing the *S* protein and those expressing the SARS-CoV receptor ACE2 [122]. But monoclonal antibodies can only recognize a single epitope, and the anti-infective effect may be poor. Finally, the development of monoclonal antibodies requires a certain time, which is difficult to achieve in clinical application in a short time.

## Future of Treatments for SARS-CoV-2 Infections

As SARS-CoV-2 is similar to SARS-CoV, applying different kinds of chemical drugs that were effective for other SARS-CoV, may be helpful for this infection too.

Results of covalent plasma and under development vaccines soon will indicate the value of these methods. Also, a new method is growing, Vanessa Monteil et al. (https://www.cell.com/pb-assets/products/coronavirus/CELL_CELL-D-20-00739.pdf), indicated that SARS-CoV-2 spike protein straightly binds to ACE2 and they showed that the SARS-CoV-2 spike protein identifies human ACE2 with higher binding affinity than Spike from SARS-CoV.

It has been indicated that in cell culture soluble ACE2 fused to Ig or camostat mesylate which is a nonspecific protease inhibitor can inhibit infection and disease with a Pseudovirus bearing the S protein of SARS-CoV-2. High doses of camostat mesylate were indicated to partially reduce SARS-CoV-2 growth [123].

In a normal adult human lung, ACE2 is expressed firstly in alveolar epithelial type II cells and it can play a role as a viral source [124]. These cells produce surfactant and it decreases surface tension, so it prohibits alveoli from collapsing, and therefore they are important for the gas exchange function of the lung [125]. Injury of these cells would illustrate the severe lung injury confirmed in COVID-19 patients. Studies have demonstrated that ACE2 is expressed in different extrapulmonary tissues like heart, blood vessels, kidney, and intestine [126–130]. The ACE2 tissue distribution in these organs may illustrate the multi-organ dysfunction confirmed in infected cases [7, 131].

Studies indicates that human kidney and blood vessel can be easily infected, which can be significantly inhibited by hrsACE2 at the first phase of infection. But these studies have limitations, the design of their studies were based on the early stages of infection, showing that hrsACE2 can block the first entry of SARS-CoV-2 infections in host cells. As such, we cannot make any predictions to the effect of hrsACE2 in later stages of the disease process (https://www.cell.com/pb-assets/products/coronavirus/CELL_CELL-D-20-00739.pdf). We recommend that further studies are needed to clarify the effect of hrsACE2 at later stages of infection in vitro and in vivo.

## Finally the Best Way for Keeping Away from COVID-19

Based on general advice of WHO for the public on 29 April 2020 people can do some simple precautions to reduce the chance of being infected or spreading COVID-19 [132]:

- . Regularly wash hands with an alcohol-based hand rub or with soap because these components can kill viruses that may be on your hands.
- . Maintain at least 1-meter distance between yourself and others because when someone coughs, sneezes, or speaks they spray small liquid droplets from their nose or mouth which may contain a virus and if you are to close, you can breathe droplets including covid-19 if the person has the disease.
- . Avoid going to crowded places because when people come together in crowds, you are more likely to come into close contact with someone who has covid-19 and so it is more difficult to maintain social distancing.
- . Avoid touching eyes, nose, and mouth because you touch many surfaces and can pick up viruses. Once contaminated, hands can transfer the virus to your eyes, nose, or mouth and from these places, viruses can enter the body and infect you.

Stay home and self-isolate even with minor symptoms such as cough, headache, mild fever, until you recover. Avoiding contact with others will protect them from possible COVID-19 and other viruses.

## Conclusion

Till the time of writing this review, there are no specific vaccines or certain treatments for COVID-19. Many ongoing clinical trials and potential existed treatments are introduced which we also reported them here in this review, including home remedy, herbal medicine, chemical drugs, plasma therapy and also vaccines. Although, all the named principles could support the goal of improving more efficient drugs and therapeutic strategies to reduce mortality of coronavirus disease but among all the ways for treathing COVID-19, prevention is better than cure. Therefore, for keeping away from COVID-19, the best way is social distancing and observing the personal hygiene. Moreover, It is a pivotal condition for defeating the current outbreak of the COVID-19 and the contribution of all related researchers from all over the world is needing. Hope this review can help researchers to have general overview on existing methods for treating COVID-19 and to develop more efficient drugs and remedies for this deadly virus in the near future. Finally, hope this virus to be eradicated very soon.

## Data Availability

Not applicable for this study.

## Abbreviations

PHEIC: Public Health Emergency of International Concern
MERS: Middle East Respiratory Syndrome
SARS: Severe Acute Respiratory Syndrome
RT-PCR: Reverse-transcription Polymerase Chain Reaction
rRT-PCR: Reverse Real-Time PCR
CT: Computed tomography
RNA: Ribonucleic Acid
DNA: Deoxyribonucleic Acid
WHO: World Health Organization
CQ: Chloroquine
MOI: Multiplicity of Infection
EC50: half-maximal Effective Concentration
CC50: Half-Cytotoxic Concentration
HCQ: Hydroxychloroquine
HIV: Human Immunodeficiency Viruses
RAAS: Renin-Angiotensin-Aldosterone System
AT1R: Angiotensin Receptor 1
ACE: Angiotensin Converting Enzyme
ARBs: Angiotensin II receptor blockers
NK: Natural Killer
IFN-I: Type-I Interferon
cGAS: Cyclic GMP–AMP Synthase
STING: Stimulator of Interferon Genes
STAT1: Signal Transducer and Activator of Transcription 1
CEP_C30: Coronavirus Endopeptidase C30
CYP3A: Cytochrome P4503A
IFN-β1b: Interferon Beta-1b
CRP: C-reactive Protein
LDH: Lactate Dehydrogenase
CDC: Chinese Centre for Disease Control and Prevention
CEPI: Coalition for Epidemic Preparedness
S-Trimer: Subunit-trimer Vaccine
RBD: Receptor-binding Domain
ACE2: Angiotensin-converting Enzyme 2
NIGEB: National Institute of Genetic Engineering and Biotechnology
